# Risk Estimates From an Online Risk Calculator Are More Believable and Recalled Better When Expressed as Integers

**DOI:** 10.2196/jmir.1656

**Published:** 2011-09-07

**Authors:** Holly O Witteman, Brian J Zikmund-Fisher, Erika A Waters, Teresa Gavaruzzi, Angela Fagerlin

**Affiliations:** ^1^Program in Health Communication and Decision MakingCenter for Bioethics and Social Sciences in MedicineUniversity of MichiganAnn Arbor, MIUnited States; ^2^Division of General MedicineDepartment of Internal MedicineUniversity of MichiganAnn Arbor, MIUnited States; ^3^Department of Health Behavior and Health EducationSchool of Public HealthUniversity of MichiganAnn Arbor, MIUnited States; ^4^Risk Science CenterSchool of Public HealthUniversity of MichiganAnn Arbor, MIUnited States; ^5^Division of Public Health SciencesDepartment of SurgeryWashington University School of MedicineSaint Louis, MOUnited States; ^6^Leeds Institute of Health SciencesFaculty of Medicine and HealthUniversity of LeedsLeedsUnited Kingdom; ^7^Department of Developmental Psychology and SocializationUniversity of PadovaPadovaItaly; ^8^VA Ann Arbor Center for Clinical Management ResearchAnn Arbor, MIUnited States; ^9^Department of PsychologyUniversity of MichiganAnn Arbor, MIUnited States

**Keywords:** Risk, risk assessment, communication, risk communication, perception, risk perception, calculators, programmable, risk calculator, Internet, online

## Abstract

**Background:**

Online risk calculators offer different levels of precision in their risk estimates. People interpret numbers in varying ways depending on how they are presented, and we do not know how the number of decimal places displayed might influence perceptions of risk estimates.

**Objective:**

The objective of our study was to determine whether precision (ie, number of decimals) in risk estimates offered by an online risk calculator influences users’ ratings of (1) how believable the estimate is, (2) risk magnitude (ie, how large or small the risk feels to them), and (3) how well they can recall the risk estimate after a brief delay.

**Methods:**

We developed two mock risk calculator websites that offered hypothetical percentage estimates of participants’ lifetime risk of kidney cancer. Participants were randomly assigned to a condition where the risk estimate value rose with increasing precision (2, 2.1, 2.13, 2.133) or the risk estimate value fell with increasing precision (2, 1.9, 1.87, 1.867). Within each group, participants were randomly assigned one of the four numbers as their first risk estimate, and later received one of the remaining three as a comparison.

**Results:**

Participants who completed the experiment (N = 3422) were a demographically diverse online sample, approximately representative of the US adult population on age, gender, and race. Participants whose risk estimates had no decimal places gave the highest ratings of believability (*F*_3,3384_ = 2.94, *P* = .03) and the lowest ratings of risk magnitude (*F*_3,3384_ = 4.70, *P* = .003). Compared to estimates with decimal places, integer estimates were judged as highly believable by 7%–10% more participants (χ^2^_3_ =17.8, *P* < .001). When comparing two risk estimates with different levels of precision, large majorities of participants reported that the numbers seemed equivalent across all measures. Both exact and approximate recall were highest for estimates with zero decimals. Odds ratios (OR) for correct approximate recall (defined as being within 50% of the original estimate) were, for one decimal place, OR = 0.65 (95% CI 0.49–0.86), for two decimal places, OR = 0.70 (95% CI 0.53–0.94), and for three decimal places, 0.61 (95% CI 0.45–0.81). Exact recall showed a similar pattern, with larger effects.

**Conclusions:**

There are subtle but measurable differences in how people interpret risk estimates of varying precision. Adding decimal places in risk calculators offers little to no benefit and some cost. Rounding to the nearest integer is likely preferable for communicating risk estimates via risk calculators so that they might be remembered correctly and judged as believable.

## Introduction

Risk calculators abound online. Anyone with Internet access, a Web browser, some interest in their future health, and five minutes to spare can enter a few pieces of information about themselves and receive an assessment of their risk of human immunodeficiency virus infection [1], breast cancer [2], heart attack [3,4], diabetes [5,6], prostate cancer recurrence [7,8], or one of multiple options from a broad array of common conditions [9]. Online risk calculators have been heralded as a means to disseminate individualized risk prediction scores with an aim toward increased understanding of personal risk, improved health behavior, higher-quality decision making, and, ultimately, better population health [10].

However, questions remain about how to best design risk calculators to achieve the goals of risk communication. Different calculators vary significantly in terms of their adherence to best practices for risk communication [11], the amount of detail and clinical specificity in their questionnaires, and the level of precision that they offer in their risk assessments. For example, a 55-year-old woman might use two different Web-based calculators to check her risk of breast cancer in the next 10 years and find that, according to one, her risk is 2.1% [12] while, according to another, it is 5.05399% [13]. (See Multimedia Appendix 1 for details.)

Robust underlying models may enable calculators to give precise risk estimates. However, it is not known whether this additional precision is helpful or harmful for people using risk calculators. In other words, we *can* be more precise, but *should* we?

The importance of this question becomes apparent when one considers the complex range of challenges inherent in risk communication. Across levels of education and expertise, many people, particularly those with poor numeracy, have trouble interpreting numbers in health-risk communications [14,15] and demonstrate biased interpretations of proportions [16]. For example, people have been shown to rate a cancer as riskier when it “kills 1286 out of 10,000 people” (about 13%) than when it “kills 24.14 out of 100 people” (about 24%) [17], and to prefer a lottery that offers 5, 6, 7, 8, or 9 winning draws in 100 (5%–9%) over a lottery that offers 1 winning draw in 10 (10%) [18]. Furthermore, people respond differently to proportions and numbers with decimals depending on the presentation format—for example, percentages (25%), natural frequencies (25 in 100), and “1 in n” formats (1 in 4) [19,20].

The precision of a number, in particular, can affect how people perceive and act on numerical information. For example, home buyers offer bids closer to the asking price for houses with more precise list prices [21] and are more likely to choose a battery-powered product with a battery life of 120–180 minutes versus one with a battery life of 2–3 hours [22]. Consumers [23,24] and investors [25] exhibit purchasing behavior suggesting that many people may interpret prices with decimals by simply truncating digits beyond the decimal point.

The effects of estimate precision in health have thus far been studied by examining responses to point estimates (eg, 9%) versus ranges of estimates (eg, 5%–13%). Previous qualitative research suggested that ranges of risk estimates may be perceived as more credible than point estimates [26]. Subsequent experimental research showed that perceived risk was larger for a range than a point estimate when the estimate was expressed in text, but there was no main effect of precision on credibility [27]. The sparse, mixed results in previous research suggest that the effect of precision on believability of a risk estimate remains an open question. Further, we propose that, although ranges and number of significant figures are both used mathematically to convey precision or lack thereof, they present different questions when it comes to layperson responses. Ranges make the imprecision explicit, whereas the number of decimal places is an implicit signal. Many risk calculators give a single point estimate [11], and, prior to this experiment, the specific effects of the number of decimal places in a risk estimate had not been studied.

In this study, we aimed to isolate the effects of precision—that is, number of decimal places—on people’s interpretations of risk estimates offered by online risk calculators. We selected believability (“How believable is this number?”) and risk magnitude (“How large or small does this number feel to you?”) as primary outcomes. Perceptions of believability and risk magnitude are critical to changing health attitudes and behavior [28], common goals of risk calculators, and we suggest that these are a reasonable first line of consideration when examining responses to a risk estimate. People are unlikely to ponder whether a risk is modifiable and what actions they might take if they lack confidence in the estimate in the first place, and a key measure for risk communication is the resulting subjective feeling of how large or small the risk is.

## Methods

### Design of Experiment

Participants were asked to imagine they were visiting a kidney cancer risk calculator. (See Multimedia Appendix 2 for full methodological details, including exact wording used, screenshots of the two mock risk calculators participants were sent to, and rationale for design choices described here.) The calculator questions used actual risk factors for kidney cancer, but randomly assigned each participant a risk estimate around 2%, slightly above the average lifetime risk statistic for US adults of 1.49% [29]. Risk estimates were expressed as integers with zero decimal places (2%), to one decimal place (1.9% or 2.1%), two decimal places (1.87% or 2.13%), or three decimal places (1.867% or 2.133%). As shown by the numbers used, numerical values either fell or rose slightly below or above 2% with increasing decimals; participants were randomly assigned to either the “falling” or “rising” group. Participants were also randomly assigned to either a shorter version (fewer questions) or longer version (more questions) of the mock risk calculator to test whether a longer questionnaire might reasonably be seen as providing a more credible estimate.

After completing the questions in the risk calculator, participants were shown the “result” that they had been randomly assigned. They were then asked to indicate the believability of the risk, how large or small it felt to them, and a series of secondary assessments about how well or poorly the following adjectives described the estimate they were given: accurate, precise, exact, likely to be wrong, scientific, and uncertain. These secondary assessments were taken from previous work done by our research group comparing point estimates and ranges [30], and were intended to collect exploratory data that might help unpack any differences found in primary outcomes.

To mimic a plausible response to receiving a risk estimate—namely, seeking a second estimate to confirm or contradict the first—participants were then directed to a second mock calculator that presented a second risk estimate. The second estimate was randomly assigned from the other three numbers in their rising or falling group of numbers. For example, participants assigned to the falling group might receive a first risk estimate of 1.9%, and their second risk estimate would be randomly assigned as either 2%, 1.87%, or 1.867%. Participants were then asked to compare the two numbers in terms of believability, as well as the secondary outcomes of accurate, precise, exact, likely to be wrong, scientific, and uncertain. To remove the possibility that recall differences might contaminate the comparisons, all comparison questions were presented with the estimates as labels, with the first estimate as the label for the first column, and the second estimate for the second column. We did not ask participants to compare the estimates in terms of risk magnitude because we predicted that the difference in expressed values (for example, 2 > 1.9) would dominate any effects of the level of precision and we therefore saw little benefit in increasing respondent burden by adding another comparison task.

**Figure 1:**
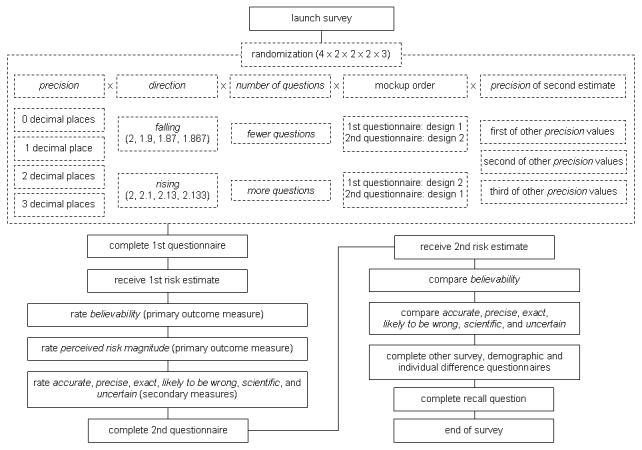
Flow Diagram of Experiment.

Participants completed another survey about hypothetical treatment choices for colon cancer, in which participants were cross-randomized to avoid any systematic interaction between the two surveys. They then completed a brief set of demographic and individual difference measures. Finally, on the last page of the combined survey, we asked participants to recall to the best of their ability both risk estimates they had been given. (The experimental procedure is detailed in Figure 1 and Multimedia Appendix 3.)

### Recruitment

Email invitations were sent to a random sample of US adults aged 30 to 70 years, selected from a panel of Internet users administered by Survey Sampling International (Shelton, CT, USA) and stratified by gender, age, and race to ensure demographic diversity. The survey did not collect identifying information. Survey Sampling International uses a complex digital fingerprinting technique to ensure respondent uniqueness [31]. The design was approved by the University of Michigan Medical Institutional Review Board.

### Measures

#### Independent Variables

*Precision* was operationalized by the number of decimals shown in the risk estimate (ie, 0, 1, 2, or 3).

The *direction* of the risk estimate refers to whether the numeric value of the estimates rose or fell with increasing precision. The *rising* condition used the numbers 2, 2.1, 2.13, and 2.133. The *falling* condition used 2, 1.9, 1.87, and 1.867.

The *number of questions* in the risk calculator was operationalized as either 4 pages (screens) of questions in the mocked-up questionnaire or the same 4 plus an additional 3 pages. The former, which contained 11 questions in all, represents the condition *fewer questions* and the latter, which contained 17 questions in all, represents the condition *more questions*.

#### Primary Outcome Variables

*Believability* of the risk estimate was assessed by asking participants to respond to the question, “Think about the risk number you were given. In your opinion, how **believable** is this number?” (emphasis original) on a 6-point Likert scale anchored by “not at all” on one end and “extremely” on the other. Responses were not labeled with their numeric value, meaning that participants did not see any numbers, only a horizontal visual array of equally spaced radio buttons. Believability was collected immediately after viewing the risk estimate, on its own survey page. For examining and reporting distribution of responses, we defined the bottom two points of the scale as representing *low believability*, the middle two points as representing *moderate believability*, and the top two points as representing *high believability*.

*Perceived risk magnitude* was a measure of how big the risk estimate felt to participants. This outcome was assessed by the question, “How **large or small** does the risk **feel** to you?” (emphasis original). Responses were collected using a horizontal slider labeled by “extremely small” on the left and “extremely large” on the right. The slider marker was visible throughout the interaction, with its default position in the center. We anticipated that, while this might anchor results around the center of the scale, such anchoring would not interfere with our analysis, which aimed only to compare slider positions between conditions. Therefore, the increased usability of making the slider marker visible was worth the potential global bias. As with the believability scale, participants saw only the visual position of the slider, not the numeric values representing their response. Values were stored to 2 decimal places on the interval [0, 1]. Risk magnitude was collected on its own survey page.

#### Secondary Outcome Variables

Secondary outcomes *accurate*, *precise*, *exact*, *likely to be wrong*, *scientific*, and *uncertain* were assessed via the questions, “We would also like to know, in your opinion: How **accurate** is this number? How **precise** is this number? How **exact** is this number? How **scientific** is this number? How **likely** is this number **to be wrong**? How **uncertain** is this number?” (emphases original). All were collected using 6-point Likert scales anchored by “not at all” on one end and “extremely” on the other, with no numeric feedback. These variables were collected together on one survey page, in a randomly ordered list.

To elicit *comparisons* between the two given risk estimates, participants were first asked to compare the two numbers in terms of believability, indicating whether the first estimate they were given or the second was more believable, or if they were equal in this respect. This comparison question was completed on its own survey page, on which the response options were labeled with the first risk estimate, then the second, then the label “both equal.” On the next page, participants were given a similar comparison task, this time applied to the same secondary outcomes used earlier: accurate, precise, exact, likely to be wrong, scientific, and uncertain, once again randomly ordered in a list.

**Figure 2:**
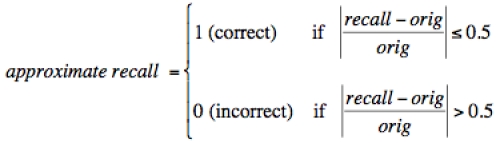
Equation for calculating participants’ approximate recall (within 50% error) of their estimated risk: *recall* represents the recalled number and *orig* represents the original given estimate.

Measures of *recall* were elicited by asking participants, “To the best of your ability, can you tell us the lifetime risk of kidney cancer from the website calculators earlier in this survey? Please enter the most detailed numbers you can remember.” We defined correct *exact recall* as a recalled number that matched the given risk estimate perfectly. We defined correct *approximate recall* as a recalled estimate that was within 50% error of the given number (Figure 2).

This definition enables a wide margin of error, which we deemed appropriate for such a small risk estimate. Thus, recall estimates between approximately 1% and 3% were defined as correct approximate recall, whereas those outside the defined range were defined as incorrect.

#### Individual Difference Measures

Individual difference measures used in this study were as follows: *subjective numeracy* [32], *cancer fear* (using an adapted breast cancer fear scale [33] that has been adapted as general cancer fear in other research [34]), and three Big-5 personality dimensions, *neuroticism*, *openness*, and *agreeableness*, which were collected using a brief index of all five dimensions [35]. We also posed an ad hoc self-assessed measure of *susceptibility to marketing*: “How much do you think your choices about the things you buy are influenced by marketing?” assessed on a 6-point Likert scale anchored by “not at all influenced” and “extremely influenced.” All of these measures were selected for analysis due to their possible moderating effect on individuals’ responses to different levels of precision.

### Statistical Analyses

We analyzed ratings of believability and risk magnitude for the first risk estimates via multivariate analysis of variance (MANOVA). We used three independent variables: precision (number of decimals), direction of values (rising versus falling), and number of questions (4 screens versus 7). We included all main effects and all 2-way interactions in the model and conducted post hoc tests on precision (the only independent variable with more than two levels) via the Tukey least significant difference test. We performed a second MANOVA with the same model to examine the effects of the independent variables on the secondary outcomes accurate, precise, exact, likely to be wrong, scientific, and uncertain.

To explore participants’ assessments of the comparisons between the two risk estimates, observed differences in proportions for each measure were tested via 2-tailed binomial tests. These tests were conducted only on data from participants who judged the two numbers as different on that measure.

Finally, we analyzed exact and approximate recall via repeated measures logistic regression, regressing recall on the number of decimals. We present both exact and approximate recall results, though we focus on approximate recall as the fair and practically relevant comparison. Recalling a value expressed to more decimal places exactly requires additional memory capacity, and it is unlikely that people would need to recall estimates to a high level of precision for any practical purpose.

Data were entered and analyzed in SPSS version 16.0 (IBM Corporation, Somers, NY, USA).

## Results

### Recruitment

Out of 4242 people who clicked the link to launch the survey, 4117 (97%) continued beyond the informed consent page, and 3422 (81%) completed the survey. All completed surveys were analyzed. Completion rates were consistent across experimental conditions. Characteristics of study participants are shown in Table 1. Participants’ ages ranged from 30 to 70 years old with mean age 50 (SD 11) years, 1723/3305 (52%) were female, participants were racially and ethnically diverse, and 1483/3402 participants (44%) had an associate’s degree or higher.

**Table 1:** Study participant characteristics (N = 3422)

Characteristic		
**Age (years), mean (SD)**		50 (11)
**Gender, n (%)**		
	Female	1723 (52%)
	Male	1582 (48%)
**Ethnicity, n (%)**		
	Hispanic	486 (14%)
	Middle Eastern	46 (1%)
**Race, n (%)**		
	White or Caucasian	2518 (74%)
	Black or African American	529 (16%)
	American Indian or Alaska Native	55 (2%)
	Asian or Asian American	150 (4%)
	Pacific Islander or Native Hawaiian	17 (0.5%)
	Other	167 (5%)
**Highest education level reached, n (%)**		
	None	2 (0.1%)
	Elementary school	4 (0.1%)
	Some high school, but no diploma	72 (2%)
	High school (diploma or GED^a^)	665 (19%)
	Trade school	186 (6%)
	Some college, but no degree	990 (29%)
	Associate’s degree (AA, AS, etc)	357 (11%)
	Bachelor’s degree (BS, BA, etc)	759 (22%)
	Master’s degree (MA, MPH, etc)	306 (9%)
	Doctoral/professional degree (PhD, MD, etc)	61 (2%)

^a^ General equivalency diploma.

### Statistical Analyses

The precision of the risk estimate was related to believability and perceived risk magnitude. In particular, risk estimates with zero decimals yielded the highest believability scores, with scores decreasing slightly with increasing number of decimal places (*F*_3,3384_ = 2.94, *P* = .03, partial eta squared = .003). Believability was not significantly related to the number of questions in the risk calculator. Participants whose estimates had more decimals felt that the risk magnitude was larger (*F*_3,3384_ = 4.70, *P* = .003, partial eta squared = .004), as did participants who were assigned to a risk calculator with fewer questions (*F*_3,3384_ = 5.85, *P* = .02, partial eta squared = .002). For both believability and risk magnitude, direction of values did not have a significant effect, and there were no significant interactions. See Table 2 for further details.

The distribution of believability ratings is shown in Table 3. Risk estimates with zero decimals yielded 7%–10% more participants who rated the estimate as highly believable (χ^2^_3_ = 17.8, *P* < .001).

**Table 2:** Primary outcomes

		Believability: 1 = not at all, 6 = extremely; mean (SD)^a^	Risk magnitude: 0 = extremely small, 1 = extremely large; mean (SD)^a^
**Precision (number of decimal places)**			
	0	4.35 (1.24) (reference)	.21 (.24) (reference)
	1	4.24 (1.23) (*P* = .07)	.24 (.24) (*P* = .03)
	2	4.21 (1.26) (*P* = .02)	.23 (.24) (*P* = .20)
	3	4.19 (1.22) (*P* = .006)	.26 (.25) (*P* < .001)
	Overall significance	*P* = .03	*P* = .003
**Direction of values (rising: 2, 2.1, 2.13, 2.133;falling: 2, 1.9, 1.87, 1.867)**			
	Rising	4.24 (1.24)	.24 (.24)
	Falling	4.26 (1.24)	.23 (.24)
	Overall significance	*P* = .59	*P* = .18
**Number of questions (fewer: 4 screens of questions;more: 7 screens of questions)**			
	Fewer	4.21 (1.22)	.25 (.25)
	More	4.28 (1.26)	.22 (.24)
	Overall significance	*P* = .10	*P* = .02

^a^ *P* values reported next to means for precision refer to Tukey least significant difference referenced against zero decimals.

**Table 3:** Distribution (n, %) of believability responses by precision (also see Table 4.1 in Multimedia Appendix 4)

		Low believability	Moderate believability	High believability
**Precision (number of decimal places)**				
	0	63 (7%)	353 (41%)	450 (52%)
	1	60 (8%)	378 (47%)	362 (45%)
	2	80 (9%)	389 (46%)	383 (45%)
	3	69 (8%)	440 (50%)	373 (42%)

Estimates with one decimal point were rated as the least uncertain compared to estimates with zero (*P* = .02), two (*P* = .001), or three (*P* = .001) decimal places (*F*_3,3314_ = 4.76, *P* = .003, partial eta squared = .004.) However, none of the related terms accurate, precise, exact, likely to be wrong, or scientific showed a significant difference between different numbers of decimals, which may suggest possible confusion about the meaning(s) of uncertainty, which has multiple meanings, each of which ought to line up conceptually with at least one of the other terms. If participants interpreted uncertainty as an assessment of the truth of the estimate, we would also expect differences in ratings of accuracy and likelihood of being wrong. If, on the other hand, uncertainty were to be interpreted as an assessment of precision, we would expect ratings of precision and exactitude to show differences. None of these differences were in evidence and, thus, the secondary measures did not suggest potential mechanisms to explain this finding.

Ratings of accuracy were higher in the condition with more questions (*F*_1,3314_ = 4.16, *P* = .04, partial eta squared = .001), but number of questions did not have a significant effect on any other terms. Direction of values did not have a significant effect, and there were no significant interactions.

Overall, none of the secondary measures suggested potential mechanisms to explain the primary findings.

Individual difference measures demonstrated strong main effects in the expected directions. (See Multimedia Appendix 4 for details.) However, none of the individual difference measures showed any statistically significant interactions with precision, suggesting that the effects of precision are consistent across types of users.

When comparing the first and second risk estimates they were given, large majorities of participants indicated equality across all measures (see Table 4 for a summary; see Multimedia Appendix 4 for tables detailing comparisons across combinations of number of decimals.) The minority of participants who thought the two numbers were different on one or more measures judged estimates with fewer decimals as more believable, but also less accurate, less precise, less exact, less scientific, and more uncertain.

**Table 4:** Comparisons of two risk estimates

	Percentage of participants who chose			
Which number is more	Number with fewer decimals	Both numbers equal	Number with more decimals	Significance of observed proportion of participants choosing fewer vs more decimals
Believable^a^?	11%	80%	9%	*P* = .006
Accurate^b^?	13%	70%	17%	*P* < .001
Precise^b^?	13%	62%	25%	*P* < .001
Exact^b^?	13%	63%	24%	*P* < .001
Scientific^b^?	11%	69%	20%	*P* < .001
Likely to be wrong^b^?	13%	74%	14%	*P* = .28
Uncertain^b^?	15%	72%	13%	*P* = .002

^a^ Primary comparison outcome, question presented first on its own survey page.

^b^ Secondary comparison outcomes, questions presented together on one page in random order.

After completing the questions comparing the two risk estimates, participants spent a median of 9.6 minutes (interquartile range 6.5 minutes) answering an unrelated survey before reaching the recall task, in which they were asked to recall both risk estimates they had been given earlier. Participants were not warned that they would be asked to recall the numbers.

The proportions of participants with correct recall are shown in Table 5 for both exact and approximate recall error. Errors in exact recall increased quickly with precision. The majority of participants had correct approximate recall across all levels of precision, but errors remained more common in estimates with decimal places than in those with zero decimals. The effects of precision were significant on both exact and approximate recall.

**Table 5:** Participants with correct recall

	Exact recall		Approximate recall	
Precision (number of decimals)	Correct	Odds ratio (95% CI)	Correct	Odds ratio (95% CI)
0	93%	Reference	96%	Reference
1	83%	0.36 (0.29–0.44)	94%	0.65 (0.49–0.86)
2	70%	0.17 (0.14–0.21)	95%	0.70 (0.53–0.94)
3	43%	0.06 (0.05–0.07)	94%	0.61 (0.45–0.81)
	Wald χ^2^_3_ = 1014, *P* < .001		Wald χ^2^_3_ = 12.1, *P* = .007	

## Discussion

### Principal Results

This study suggests that risk calculators that produce risk estimates with different levels of precision can result in different perceptions of those estimates in terms of believability and risk magnitude, as well as differences in recall. In this experiment, risk estimates with zero decimals were judged as the most believable. People may find integers somewhat more believable than numbers with decimals simply because integers are easier to understand. As evidenced by, for example, jokes and confusion about an average American family having 2.2 children, it is challenging for people to map population-based statistics onto individual circumstances. Indeed, many people, even those who are well educated, have trouble with probabilities and percentages [36,37]. This is particularly true of people with poor numeracy skills [38]. The fact that people have trouble with this concept is perhaps not surprising given that a patient doesn’t experience the probability of an event occurring; she or he either experiences the event or not [39]. Therefore, truly understanding a risk estimate requires conceptually mapping a probability onto a binary outcome. Adding decimals to the risk estimate only makes this task more challenging. Simplifying the risk estimate might therefore increase understanding and, hence, believability.

Risk estimates with the least precision (zero decimals) also felt smaller on average than estimates with greater precision. This finding parallels previous research on ratio bias, in which statistical frequencies presented using smaller denominators felt smaller than those that used larger denominators [15,16]. We speculate that seeing fewer numbers evokes in people a smaller degree of number sense and hence lower risk magnitudes.

Lower precision was also associated with better recall of the given risk estimates. It is not particularly surprising that people found numbers with more decimals places more difficult to remember perfectly. Recalling four digits takes considerably more cognitive capacity than recalling one. It is more notable that, even when allowing for a very generous margin of error in a recall task that took place shortly after the estimate was provided, there were statistically significant differences in approximate recall between estimates with zero decimals and all three estimates with decimals. This means that using decimals in a risk estimate not only reduces the chances that users will be able to recall the number exactly, but also reduces the likelihood that they will be able to remember it even approximately. This may be partially attributable to a lack of understanding about the meaning of decimals, because if people are unable to comprehend the data that they have been given, they will not be able to turn it into information that can later be recalled.

This study also suggests that the number of questions asked in a risk calculator may have an effect on perceived risk magnitude. People who completed a longer questionnaire judged the risk estimate as smaller. Although our study found no statistical effect of the number of questions in the calculator on believability, this may have been because even our version with fewer pages of questions was sufficient to be over a threshold of believability. Further research will be required to explore the effects of very brief questionnaires on people’s assessments of risk calculators, but it is worth noting that, even with a very detailed questionnaire, the estimate with zero decimals still garnered the highest believability scores.

### Limitations

There are three main limitations to this study. First, this experiment was based on a hypothetical scenario with artificial risk estimates all around 2%. We do not know whether similar effects would be found in situations in which numbers are real and individualized for the user, people are self-motivated to seek out the risk information, and/or numbers are larger or smaller. However, our mock risk calculator was modeled after real-world examples, and thus we have no reason to believe that patient behavior would differ when using an actual risk calculator to which he or she was directed, for example, in a routine monthly email from his or her health care group or system. We acknowledge that it is more difficult to predict how people might respond in a similar situation in which they are deliberately seeking out the information. However, conducting a controlled experiment in which the only variation was random assignment of the number of decimals in the risk estimate allowed us to control for many of the complexities of how people decide whether a piece of online health information is trustworthy, thereby isolating the unique effects of the precision of the risk estimate. Findings regarding real-world use of risk calculators will depend to some extent on users’ prior expectations regarding their risk; for example, people may be resistant to accepting risk estimates that are higher than their prior expectations [40]. We did not assess prior expectations in this study because we wished to avoid biasing responses to our questionnaire [41], so we cannot speculate on how relationships between prior expectations and assigned estimates might have influenced participants’ responses. We believe our findings will remain applicable to real-world risk calculators that display risks of varying magnitudes, but confirming this belief requires further research.

Second, all of the statistically significant findings in this study have small effect sizes. Single-digit *F* statistics, small odds ratios, and modest findings in post hoc tests suggest only small differences in the way people interpret and recall risk estimates with varying levels of decimal precision. However, online risk calculators aim to reach large numbers of people amid a cacophony of conflicting and confusing health information. Therefore, as with other challenges in the complex field of health communication, small effects may be worth attention, especially when they can be brought about by design modifications as easily implemented as rounding risk estimates to the nearest integer. If developers of a risk calculator would like users to judge received estimates as highly believable, this research suggests that expressing results as integers may help with this goal. The simplicity of this design change implies a high benefit-cost ratio.

Third, although our study included some secondary outcomes selected in the hopes that these might help unpack any differences found in the primary outcomes, effects on the secondary measures were largely absent. This may be partly attributable to the small effect sizes on the primary outcomes—it can be harder to unpack a small box. Nonetheless, it would be useful to better understand the mechanisms behind any differences in how people perceive risk estimates expressed as integers versus those with decimal places. Further research will be required to achieve this understanding.

### Comparison With Prior Work

To our knowledge, there has been no prior work examining the effect of decimal precision in risk estimates.

However, our finding that increased precision leads to lower believability is in line with previous qualitative research suggesting that ranges of risk estimates may be seen as more credible than point estimates [26]. On the other hand, our finding is in contrast with experimental work that followed this qualitative study, which found no main effect on credibility [27]. This lack of agreement may be due to the inherent difference between the way people interpret ranges versus point estimates and the way they interpret numbers of decimal places. It may also be due to measurement differences. In the previous study, credibility was defined by an ad hoc scale that combined two items—ratings of trust in the results of the computer program and perceptions of accuracy of the risk estimate—whereas in our study, we assessed believability by asking participants to rate the believability of the estimate directly.

Our finding that increased precision also leads to perception of lower risk magnitude is in contrast with previous research in which more ambiguous risk estimates, meaning those expressed as ranges rather than point estimates, led to increased risk perceptions [26,27]. We believe that this difference is another example of differences in interpretation between different ways of expressing precision—that is, decimals places, or ranges and point estimates. Both findings (those in previous work and ours) support our speculation that seeing fewer numbers may elicit a lower overall sense of magnitude.

Research in consumer pricing suggests that prices with decimal places may be interpreted by simply ignoring numbers after the decimal place. If this were to also occur in health risks, we would expect to observe an interaction between precision and direction on perceived risk magnitude. That is, we would expect risk magnitude scores for the rising condition (2, 2.1, 2.13, and 2.133) to remain consistent regardless of precision, while those for the falling condition (2, 1.9, 1.87, and 1.867) would decrease between the first estimate and the other 3. We did not observe any such interaction, and we suggest that this is likely because, even if this effect exists in the health context, it may be significantly smaller and thus not detectable in this study. In other words, a price of $1 may feel different from $2, but a 1% risk may not feel meaningfully smaller than a 2% risk.

Our finding that fewer decimal places leads to better recall is consistent with research reporting that health communications that provide less detail lead to higher comprehension than those that provide more detail [42,43].

### Conclusions

There are subtle but significant differences in how people interpret risk estimates of varying precision. Increasing precision in the form of decimal places shows no clear benefit and suggests small but significant costs. Results from our experiment suggest that, in general, rounding to the nearest integer is preferable for communicating small risk estimates so that they may be judged as believable and remembered correctly. Given these findings, we recommend that risk calculator designers structure their algorithms to express risk in integers, though expressions to 1 decimal place may also be acceptable in situations when user recall of the number is not an important consideration or when greater precision is necessary to show differences between two or more numbers.

## Multimedia Appendix 1

Breast Cancer Risk Calculator Example.

## Multimedia Appendix 2

Detailed Methods.

## Multimedia Appendix 3

Detailed Flow Diagram.

## Multimedia Appendix 4

Additional Details of Results.

## Abbreviations

MANOVA: multivariate analysis of variance
OR: odds ratio

## References

[1] (2006). AIDSGAME.com.

[2] National Cancer Institute (2008). US National Institutes of Health.

[3] Mayo Clinic staff (2010). MayoClinic.com.

[4] National Cholesterol Education Program NCEP.

[5] American Diabetes Association (2009). ADA.

[6] American Diabetes Association (2010). ADA.

[7] Prostate Cancer Canada.

[8] (2011). Memorial Sloan-Kettering Cancer Center.

[9] (2007). Siteman Cancer Center at Barnes-Jewish Hospital and Washington University School of Medicine.

[10] Holmberg C, Harttig U, Schulze MB, Boeing H (2011). The potential of the Internet for health communication: the use of an interactive on-line tool for diabetes risk prediction. Patient Educ Couns.

[11] Waters EA, Sullivan HW, Nelson W, Hesse BW (2009). What is my cancer risk? How internet-based cancer risk assessment tools communicate individualized risk estimates to the public: content analysis. J Med Internet Res.

[12] Halls SB (2008). Detailed Breast Cancer Risk Calculator.

[13] (1999). GenneX Healthcare Technologies, Inc.

[14] Nelson W, Reyna VF, Fagerlin A, Lipkus I, Peters E (2008). Clinical implications of numeracy: theory and practice. Ann Behav Med.

[15] Woloshin S, Schwartz LM, Byram S, Fischhoff B, Welch HG (2000). A new scale for assessing perceptions of chance: a validation study. Med Decis Making.

[16] Weinstein ND (1999). What does it mean to understand a risk? Evaluating risk comprehension. J Natl Cancer Inst Monogr.

[17] Yamagishi K (1997). When a 12.86% mortality is more dangerous than 24.14%: implications for risk communication. Appl Cogn Psychol.

[18] Denes-Raj V, Epstein S (1994). Conflict between intuitive and rational processing: when people behave against their better judgment. J Pers Soc Psychol.

[19] Lipkus IM (2007). Numeric, verbal, and visual formats of conveying health risks: suggested best practices and future recommendations. Med Decis Making.

[20] Cuite CL, Weinstein ND, Emmons K, Colditz G (2008). A test of numeric formats for communicating risk probabilities. Med Decis Making.

[21] Janiszewski C, Uy D (2008). Precision of the anchor influences the amount of adjustment. Psychol Sci.

[22] Zhang CYZ, Schwarz N (2011). The granularity effect. Proceedings.

[23] Schindler RM, Kirby PN (1997). Patterns of rightmost digits used in advertised prices: implications for nine-ending effects. Journal of Consumer Research.

[24] Stiving M (2000). Price-endings when prices signal quality. Management Science.

[25] Sonnemans J (2006). Price clustering and natural resistance points in the Dutch stock market: a natural experiment. European Economic Review.

[26] Han PK, Klein WM, Lehman TC, Massett H, Lee SC, Freedman AN (2009). Laypersons' responses to the communication of uncertainty regarding cancer risk estimates. Med Decis Making.

[27] Han PK, Klein WM, Lehman T, Killam B, Massett H, Freedman AN (2011). Communication of uncertainty regarding individualized cancer risk estimates: effects and influential factors. Med Decis Making.

[28] Weinstein ND (2000). Perceived probability, perceived severity, and health-protective behavior. Health Psychol.

[29] Altekruse SF, Kosary CL, Krapcho M, Neyman N, Aminou R, Waldron W, Ruhl J, Howlader N, Tatalovich Z, Cho H, Mariotto A, Eisner MP, Lewis DR, Cronin K, Chen HS, Feuer EJ, Stinchcomb DG, Edwards BK (2010). SEER Cancer Statistics Review 1975-2007.

[30] Fuhrel-Forbis A, Zikmund-Fisher BJ, Exe N, Kahn V, Witteman H, Jankovic A, Angott AM, Ubel PA (2011). Imprecise definitions of "precise": preferences for precision depend on context.

[31] SSI Survey Sampling International (2010). SSI.

[32] Fagerlin A, Zikmund-Fisher BJ, Ubel PA, Jankovic A, Derry HA, Smith DM (2007). Measuring numeracy without a math test: development of the Subjective Numeracy Scale. Med Decis Making.

[33] Champion VL, Skinner CS, Menon U, Rawl S, Giesler RB, Monahan P, Daggy J (2004). A breast cancer fear scale: psychometric development. J Health Psychol.

[34] Miles A, Voorwinden S, Mathews A, Hoppitt LC, Wardle J (2009). Cancer fear and the interpretation of ambiguous information related to cancer. Cognition & Emotion.

[35] Gosling SD, Rentfrow PJ, Swann WB (2003). A very brief measure of the Big-Five personality domains. Journal of Research in Personality.

[36] Lipkus IM, Samsa G, Rimer BK (2001). General performance on a numeracy scale among highly educated samples. Med Decis Making.

[37] Hoffrage U, Lindsey S, Hertwig R, Gigerenzer G (2000). Communicating statistical information. Science.

[38] Peters E, Västfjäll D, Slovic P, Mertz CK, Mazzocco K, Dickert S (2006). Numeracy and decision making. Psychol Sci.

[39] Walker VR (1995). Direct Inference, Probability, and a Conceptual Gulf in Risk Communication. Risk Analysis.

[40] Harle CA, Downs JS, Padman R (2011). A clustering approach to segmenting users of internet-based risk calculators. Methods Inf Med.

[41] Fagerlin A, Zikmund-Fisher BJ, Ubel PA (2005). How making a risk estimate can change the feel of that risk: shifting attitudes toward breast cancer risk in a general public survey. Patient Educ Couns.

[42] Zikmund-Fisher BJ, Fagerlin A, Ubel PA (2010). A demonstration of ''less can be more'' in risk graphics. Med Decis Making.

[43] Peters E, Dieckmann N, Dixon A, Hibbard JH, Mertz CK (2007). Less is more in presenting quality information to consumers. Med Care Res Rev.

