# Supplementary material for: Risk Estimates From an Online Risk Calculator Are More Believable and Recalled Better When Expressed as Integers
Source: J Med Internet Res. 2011 Sep 7;13(3):e54. doi: 10.2196/jmir.1656 (PMC3222170; doi:10.2196/jmir.1656)
Supplement: Supplementary file 1 [file jmir_v13i3e54_app1.pdf]

Breast Cancer Risk Calculator Example

Risk calculator 1 input:

55 years old
white
no sisters, daughters or mother with breast cancer
no breast biopsies
began her menstrual cycles at age 13
had her first child at age 23

http://www.halls.md/breast/risk.htm

Risk calculator 2 input:

55 years old
white
maternal aunt with breast cancer

http://www.estronaut.com/a/breastInteractive2.htm
