# Supplementary material for: Risk Estimates From an Online Risk Calculator Are More Believable and Recalled Better When Expressed as Integers
Source: J Med Internet Res. 2011 Sep 7;13(3):e54. doi: 10.2196/jmir.1656 (PMC3222170; doi:10.2196/jmir.1656)
Supplement: Supplementary file 2 [file jmir_v13i3e54_app2.pdf]

Detailed Methods

Design of experiment

Participants were asked to imagine they were visiting a kidney cancer risk calculator. (See below for exact wording used.) We used kidney cancer as the disease context because we believed that most people would have a sense of what cancer is and recognize kidneys as a body part, but would not have detailed knowledge about kidney cancer *per se*. As a result, we believed that participants would be unlikely to have prior numerical expectations for their risk of kidney cancer, as compared to more well-publicized risk estimates for cancers such as breast and prostate.

Text used in explanations of risk calculators

Text participants saw before proceeding to first mockup calculator:

"Imagine that you visit a web page created by a prominent, well-respected university's medical school.

The researchers at this medical school have created a web-based calculator that gives you your personal lifetime risk of kidney cancer.

Your lifetime risk is the chance that you will develop kidney cancer at some point during your life.

Please use the calculator on the following pages. After that, we will ask you some questions.

Note: We are not sending you to a new website. We are just using the other website's design. You will not be able to click most of the links. You will only be able to answer the questions and click 'Next' to proceed."

Text participants saw before proceeding to second risk calculator:

"Now imagine that you visit a second website with a cancer risk calculator, this one belonging to a different, but equally well-respected, university's medical school.

This calculator uses some of the same questions as the previous one, so we have copied your answers wherever possible to save you the trouble of re-entering them.

Some previous questions are not included in this calculator, and there are a few new questions, instead. Please answer the new questions and click 'Next' to see your results from this other calculator.

Again, we are not sending you to a new website; we are just using the other website's design. You will not be able to click most of the links. You will only be able to answer the questions and click 'Next' to proceed."

To devise the calculator questions, we used actual risk factors for kidney cancer. However, input from participants did not influence the risk estimate they were given in any way – all participants received the same lifetime risk estimate rounded to different levels of precision. A disclaimer at the conclusion of the survey informed participants that the scenario was completely hypothetical and the risk number they had been given was not real. The risk estimate assigned was set at 2%, slightly above the average lifetime risk statistic for U.S. adults of 1.49% [29]. We rounded the estimate up to 2% rather than down to 1% in order to avoid any potential effects that might apply only to numbers beginning with zeroes.

Participants were randomized by three factors. First, they were assigned to one of two groups of lifetime risk estimates. All estimates rounded to 2% but values either fell slightly with more decimals (2, 1.9, 1.87, 1.867) or rose slightly (2, 2.1, 2.13, 2.133.) We included this factor to ensure that comparisons of outcomes between different levels of precision were not a function of comparing smaller numbers to larger, or vice versa. Digits used in decimals places were selected according to research judgment, with the goals of minimizing possible perceptions of magnitude differences between estimates while also balancing the use of different digits. Second, within each group, they were randomized to receive their first risk estimate with one of four levels of precision (zero, one, two, or three decimal places). We predicted that precision might be important to users' assessments of believability and risk magnitude (how large or small the number feels to them) given that prior work has suggested relationships between other manifestations of precision, believability [26, 27] and risk magnitude [27]. Third, they were also randomly assigned to either a shorter version (fewer questions) or longer version (more questions) of the mock risk calculator. This third factor was included to account for the fact that existing risk calculators can vary both in the number of questions in their questionnaire and level of precision of the risk estimate they offer. We predicted that this factor might be important to users' assessments of believability and risk magnitude (how large or small the number feels to them) because a longer questionnaire might reasonably be seen as providing a more credible estimate and the time invested in answering a longer survey could lead to either a heightened or lowered sense of being at risk.

After completing the questions in the risk calculator, participants were shown the "result" that they had been randomly assigned. They were then asked to indicate the believability of the risk, how large or small it felt to them, and a series of secondary assessments about how well or poorly the following adjectives described the estimate they were given: accurate, precise, exact, likely to be wrong, scientific, and uncertain. These secondary assessments were taken from previous work done by our research group comparing point estimates and ranges [30], and were intended to collect exploratory data that might help unpack any differences found in primary outcomes.

Participants were then asked to imagine that, after receiving this risk estimate, they visited another kidney cancer lifetime risk calculator in order to see what number it might

provide. This was intended to mimic a plausible response to receiving a risk estimate, namely, seeking a second estimate to confirm or contradict the first. For this reason, we designed two mock web-based risk calculators, hosted by fictitious universities (see Figures 1 and 2.) Participants were randomized to see one calculator for their first risk estimate and the other calculator for the second (slightly different) risk estimate. To help make the difference plausible, participants were told that the second calculator used some of the same questions as the first, and that their responses had been carried over. However, there were some questions that were different, and they would be asked to complete these questions that they had not been already asked. After completing those questions, they viewed a second risk estimate. For their second estimate, participants were randomized to receive one of the other three numbers in their rising or falling group of numbers. For example, participants assigned to the "falling" group might receive a first risk estimate of 1.9%, and their second risk estimate would be randomly assigned as either 2%, 1.87%, or 1.867%. Participants were then asked to compare the two numbers in terms of believability as well as the secondary outcomes accurate, precise, exact, likely to be wrong, scientific, and uncertain.

Figure 1: Mock website

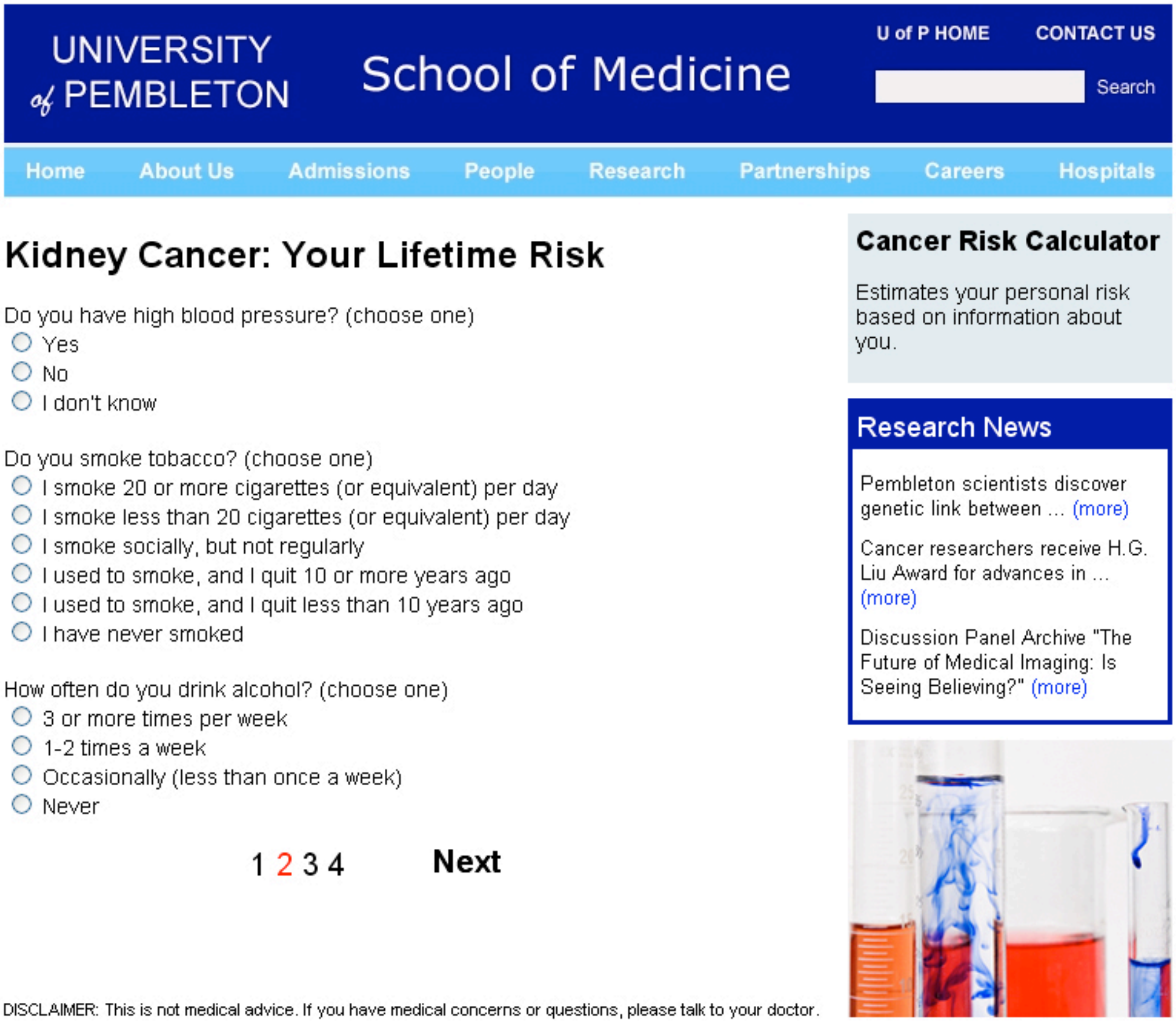

Figure 2: Mock website

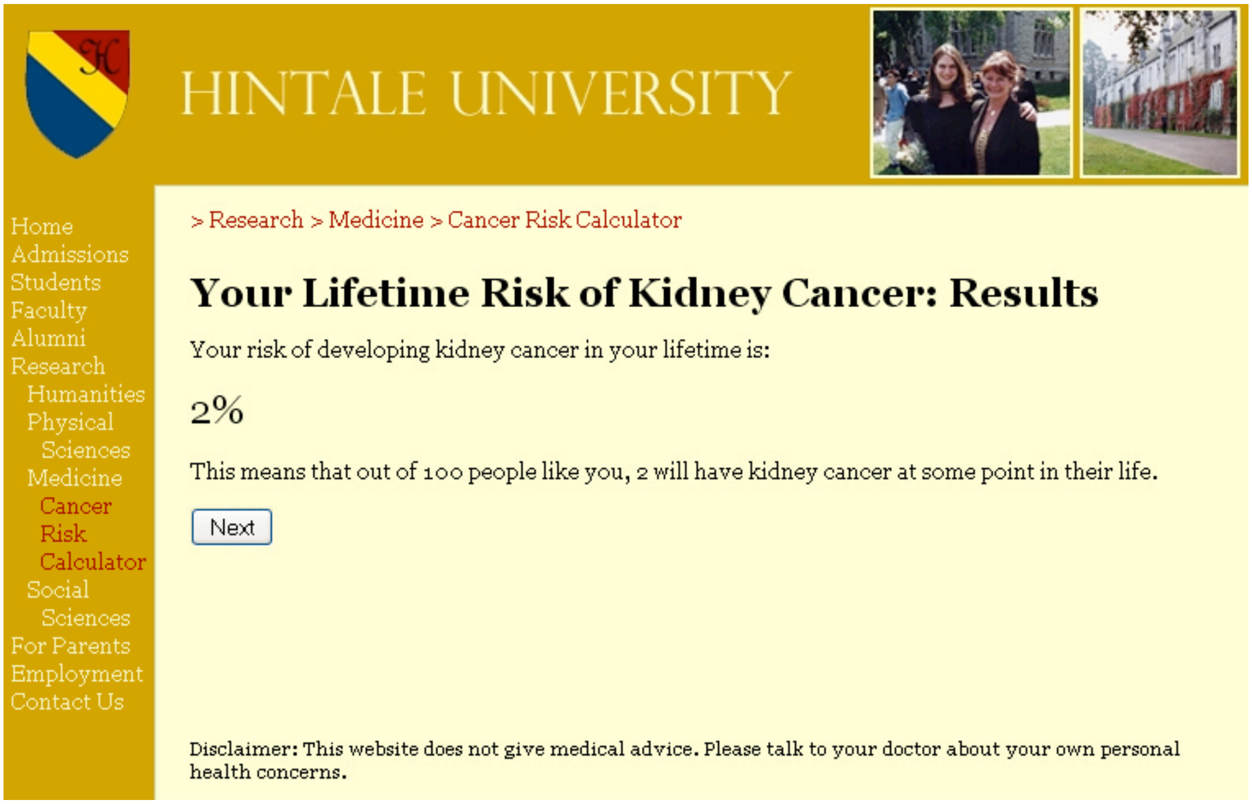

Figure 3: Flow Diagram of Experiment (See Multimedia Appendix 3: Detailed Flow Diagram for detailed version.)

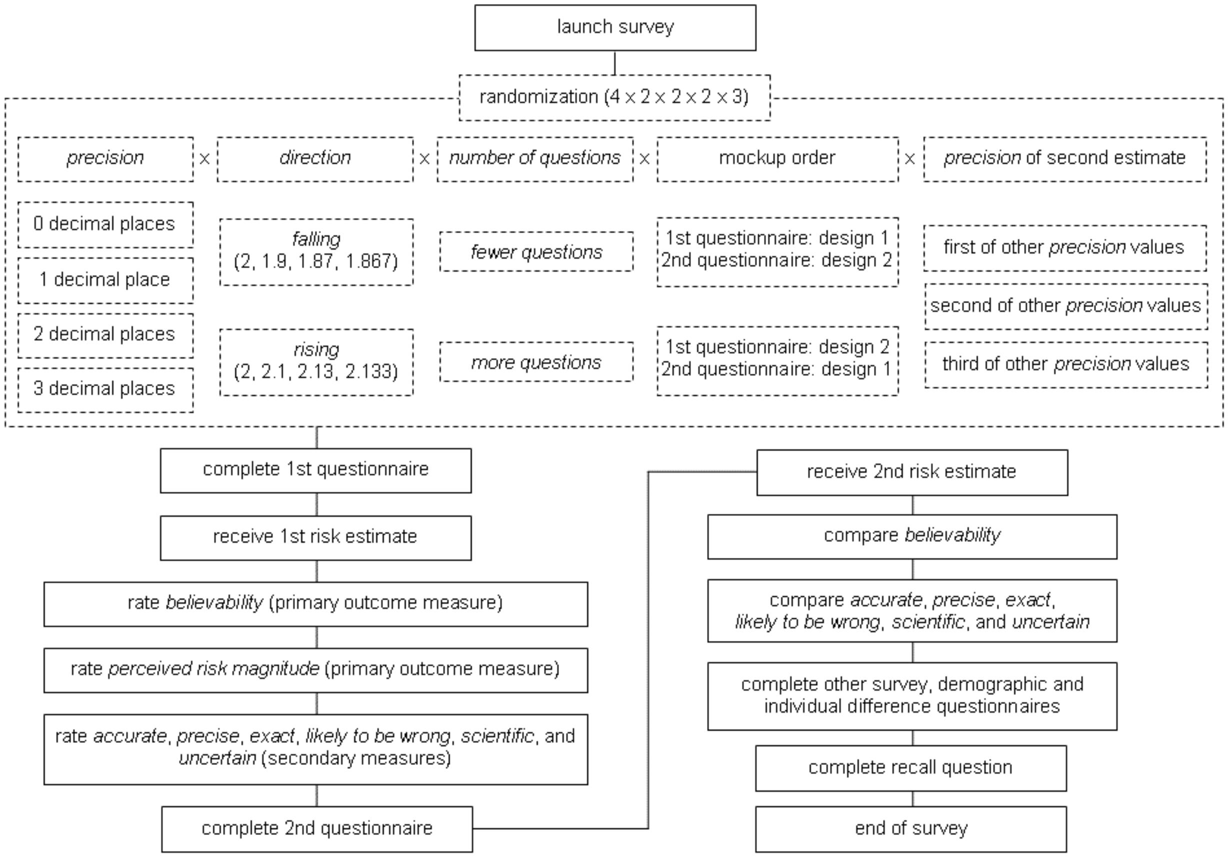

In order to remove the possibility that recall differences might contaminate the comparisons, all comparison questions were presented with the estimates as labels, with the first estimate as the label for the first column, and the second estimate for the second column (see Figure 4.) We did not ask participants to compare the estimates in terms of risk magnitude because we predicted that the difference in expressed values (for example, 2 > 1.9) would dominate any effects of the level of precision and therefore saw little benefit in increasing respondent burden by adding another comparison task.

Figure 4: Comparison of Estimates

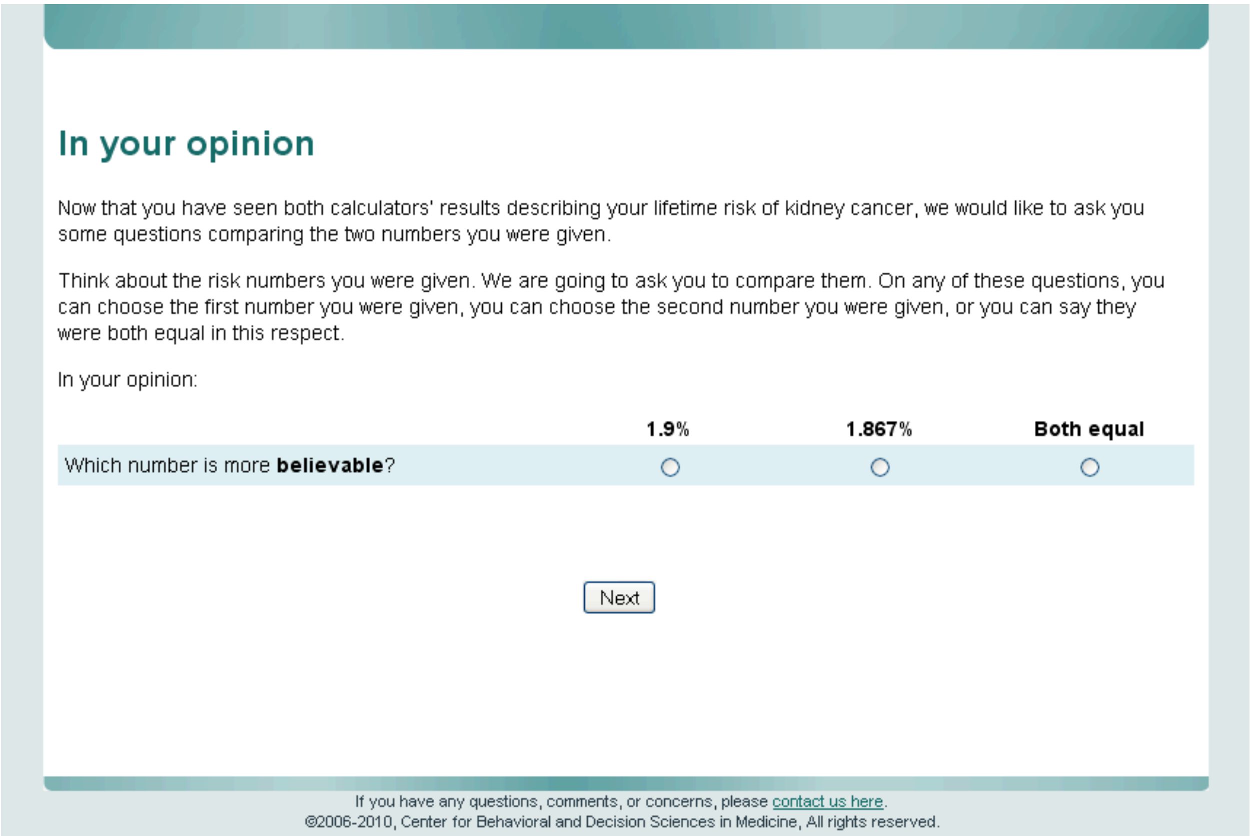

Finally, after participants completed another survey about hypothetical treatment choices for colon cancer, in which participants were cross-randomized to avoid any systematic interaction between the two surveys, and a brief set of demographic and individual difference measures, on the last page of the combined survey, we asked participants to recall to the best of their ability both risk estimates they had been given.

Participants were not offered a chance to go back and change their answers. We did not make any attempt to block their browser's Back button, meaning that if they were motivated to do so, they could paginate back at any time. However, we retained only their initial answers in our database for analysis. In total, depending on study arm randomization in this study (decimals) and participant responses to two questions in the other study (colon cancer), participants saw between 37 and 41 brief pages of survey questions. Including demographic questions, the total number of response items ranged from 90 to 99.

Participants were invited to contact the researchers at any time via a link at the bottom of each survey page, and, at the end of the brief survey, participants were reminded that the experiment was hypothetical, that we do not know their real risk for kidney cancer, and that, should they have health concerns, they should consult with their health care provider.

Recruitment

Email invitations were sent to a random sample of U.S. adults aged 30 to 70, selected from a panel of Internet users administered by Survey Sampling International (SSI) and stratified by gender, age, and race to ensure demographic diversity. See below for survey announcement email. The number of email invitations sent to each stratum was dynamically adjusted to maintain demographic balance despite varying response rates. Invitations were sent and data collected between June 9 and June 18, 2010. Participants who clicked on the invitation to take the voluntary survey were sent directly to the survey's URL within the website of the research center hosting the survey at the University of Michigan (cbdsm.org.) The survey was not visually embedded in the site (see Figure 5 below), meaning that the user was not directed to the research center's larger website at any point in the survey. The survey did not collect identifying information. SSI uses a complex digital fingerprinting technique to ensure respondent uniqueness [31]. All participants who completed the survey were entered into both an instant-win contest and a drawing administered by SSI every quarter for modest prizes. The odds of winning the drawing were approximately 1,650,000:1. Participants in subgroups that traditionally have lower response rates, namely, people who had been previously identified as Asian, African-American or Hispanic, also received a small ($3) incentive for completing the survey. The design was approved by the University of Michigan Medical Institutional Review Board.

Survey announcement emails

Email survey invitation

NOTE: Asian, African-American, and Hispanic subjects were offered an additional 300 points incentive as per the standard practice of SSI. These subgroups have lower response rates and this ensures we have adequate representation from these subgroups.

SUBJECT: <Earn Three Hundred Points>: Research on Medical Decisions

FROM: SurveySpot

Details of today's survey:

Topic: The University of Michigan is asking for your participation in an important research study that will help researchers learn valuable information about how people feel about health risks and make decisions about health conditions. Some of our questions will require time and thought, but your answers will be very important to help researchers understand how people make medical choices.

Reward: If you qualify and complete this survey, you will receive <300 points,> an Instant Win game play, and an entry into the $25,000 sweepstakes.

Survey length: About 15 minutes.

Click this link to start: URL

Your participation is important to us. Thank you for taking part in this research study.

Questions? Please reference survey number ###

Reminder Emails

SUBJECT: <Earn Three Hundred Points:> Research on Medical Decisions

FROM: SurveySpot

Reminder! You have been invited to participate in the following survey:

Topic: The University of Michigan is asking for your participation in an important research study that will help researchers learn valuable information about how people make decisions about important health conditions. Some of our questions will require time and thought, but your answers will be very important to help researchers understand how people make medical choices.

Reward: If you qualify and complete this survey, you will receive <300 points,> an Instant Win game play, and an entry into the $25,000 sweepstakes.

Survey length: About 15 minutes.

Click this link to start: URL

Your participation is important to us. Thank you for taking part in this research study.

Questions? Please reference survey number ###

The survey was programmed on the research center's custom survey engine, which is used to run surveys approximately once or twice per month. After having been reviewed by research colleagues, the survey was pre-tested for functionality, clarity and usability in a convenience sample of personal contacts. When launching the questionnaire, we first sent two small sets of email invitations (each representing 2% of our target population) one day apart to confirm our estimated time to completion and to look for signs of unusual responses, functionality problems, or unexpected dropout rates. We observed no such issues, and launched larger amounts on subsequent days.

Figure 5: Survey Introduction and Informed Consent Page

Introduction

Thank you for your time.

This study is being conducted by the Center for Behavioral and Decision Sciences in Medicine at the University of Michigan.

By taking this survey, you can help researchers understand the best ways to present medical information to the public.

This survey will take about 10-15 minutes. Your name will not be recorded anywhere in this survey. All of your answers will be completely anonymous. If you choose to complete the survey, you can stop at any time. You may skip any questions you don't want to answer. If you want to stop and restart the survey at a later time, just click on the survey link in your email invitation again to continue where you left off.

If you have any questions about this survey, please click on the link that appears at the bottom of each page.

This study was approved by the Medical Institutional Review Board. If you have any concerns, please contact the Board at:

> IRBMED
> 517 W. William, Argus I
> Ann Arbor, MI 48103-4943
> (734)763-4768
> irbmed@umich.edu
> Ref# Ame00017870

Your help means a lot to us. We thank you again for taking the time to complete this survey.

Next

If you have any questions, comments, or concerns, please contact us here.

©2006-2010, Center for Behavioral and Decision Sciences in Medicine, All rights reserved.
