# Supplementary figures and images for: Risk Estimates From an Online Risk Calculator Are More Believable and Recalled Better When Expressed as Integers
Source: J Med Internet Res. 2011 Sep 7;13(3):e54. doi: 10.2196/jmir.1656 (PMC3222170; doi:10.2196/jmir.1656)

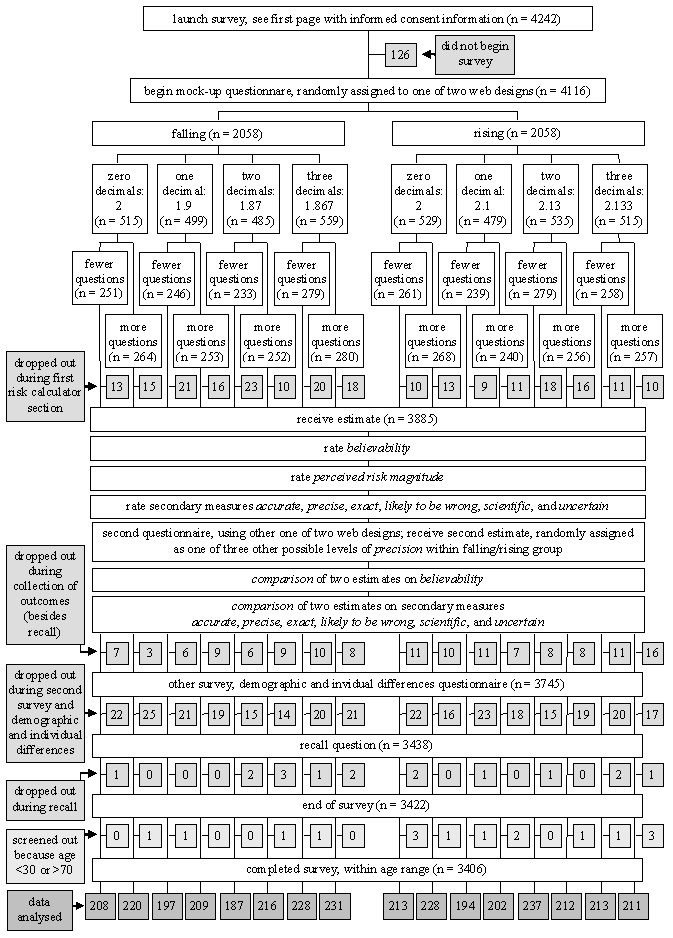

Supplement: Supplementary file 3 [file jmir_v13i3e54_app3.png]
