# Supplementary material for: Risk Estimates From an Online Risk Calculator Are More Believable and Recalled Better When Expressed as Integers
Source: J Med Internet Res. 2011 Sep 7;13(3):e54. doi: 10.2196/jmir.1656 (PMC3222170; doi:10.2196/jmir.1656)
Supplement: Supplementary file 4 [file jmir_v13i3e54_app4.pdf]

Additional Details of Results

Table 4.1: Believability Responses by Precision

		believability, N (%)					
		1 = not at all	2	3	4	5	6 = extremely
Precision (number of decimal places)	0	27 (3%)	36 (4%)	143 (17%)	210 (24%)	300 (35%)	150 (17%)
	1	22 (3%)	38 (5%)	154 (19%)	224 (28%)	236 (30%)	126 (16%)
	2	24 (3%)	56 (7%)	158 (19%)	231 (27%)	244 (29%)	139 (16%)
	3	27 (3%)	42 (5%)	174 (20%)	266 (30%)	240 (27%)	133 (15%)

Tables 4.2 - 4.7: Comparison Tables

	which is more **believable**?		
comparison	fewer decimals	equal	more decimals
0 decimals vs. 1 decimal	11%	78%	10%
0 decimals vs. 2 decimals	13%	74%	13%
0 decimals vs. 3 decimals	15%	75%	10%
1 decimal vs. 2 decimals	12%	81%	7%
1 decimal vs. 3 decimals	10%	80%	9%
2 decimals vs. 3 decimals	6%	89%	5%
overall	11%	80%	9%

	which is more **accurate**?		
comparison	fewer decimals	equal	more decimals
0 decimals vs. 1 decimal	12%	72%	16%
0 decimals vs. 2 decimals	17%	63%	20%
0 decimals vs. 3 decimals	17%	62%	21%
1 decimal vs. 2 decimals	14%	74%	11%
1 decimal vs. 3 decimals	12%	70%	18%
2 decimals vs. 3 decimals	9%	78%	13%
overall	13%	70%	17%

	which is more **precise**?		
comparison	fewer decimals	equal	more decimals
0 decimals vs. 1 decimal	12%	69%	20%
0 decimals vs. 2 decimals	16%	56%	28%
0 decimals vs. 3 decimals	14%	51%	35%
1 decimal vs. 2 decimals	14%	67%	19%
1 decimal vs. 3 decimals	10%	59%	28%
2 decimals vs. 3 decimals	10%	72%	19%
overall	13%	62%	25%

	which is more **exact**?		
comparison	fewer decimals	equal	more decimals
0 decimals vs. 1 decimal	12%	69%	20%
0 decimals vs. 2 decimals	15%	57%	28%
0 decimals vs. 3 decimals	17%	52%	32%
1 decimal vs. 2 decimals	14%	68%	18%
1 decimal vs. 3 decimals	12%	62%	26%
2 decimals vs. 3 decimals	11%	71%	18%
overall	13%	63%	24%

	which is more **scientific**?		
comparison	fewer decimals	equal	more decimals
0 decimals vs. 1 decimal	10%	73%	17%
0 decimals vs. 2 decimals	14%	62%	24%
0 decimals vs. 3 decimals	11%	61%	28%
1 decimal vs. 2 decimals	12%	75%	12%
1 decimal vs. 3 decimals	10%	70%	20%
2 decimals vs. 3 decimals	9%	76%	15%
overall	11%	69%	20%

	which is more likely to be wrong*?		
comparison	fewer decimals	equal	more decimals
0 decimals vs. 1 decimal	12%	76%	12%
0 decimals vs. 2 decimals	17%	66%	17%
0 decimals vs. 3 decimals	16%	67%	17%
1 decimal vs. 2 decimals	9%	76%	15%
1 decimal vs. 3 decimals	12%	77%	11%
2 decimals vs. 3 decimals	9%	82%	9%
overall	13%	74%	14%

*no significant difference

	which is more **uncertain**?		
comparison	fewer decimals	equal	more decimals
0 decimals vs. 1 decimal	13%	76%	11%
0 decimals vs. 2 decimals	20%	63%	17%
0 decimals vs. 3 decimals	19%	66%	16%
1 decimal vs. 2 decimals	14%	74%	12%
1 decimal vs. 3 decimals	16%	72%	12%
2 decimals vs. 3 decimals	11%	80%	9%
overall	15%	72%	13%

Details of Results for Individual Difference Measures

People with lower subjective numeracy perceived risk estimates as less believable ($F(1, 3340) = 164.88$, $P=.001$, partial eta squared = .982) and larger in magnitude ($F(1, 3340) = 68.20$, $P=.004$, partial eta squared = .958) than those with higher subjective numeracy. People who scored higher on cancer fear gave higher believability ($F(1, 3340) = 43.57$, $P=.006$, partial eta squared = .934) and risk magnitude ratings ($F(1, 3340) = 92.85$, $P=.002$, partial eta squared = .969) than those who scored lower. Self-assessments of susceptibility to marketing demonstrated good self perceptions: people who said they were more susceptible to marketing gave higher believability ($F(1, 3340) = 69.21$, $P=.004$, partial eta squared = .958) and risk magnitude ratings ($F(1, 3340) = 39.33$, $P=.008$, partial eta squared = .929) than those who judged themselves as less susceptible. Finally, people who scored high on openness gave higher believability ratings ($F(1, 3340) = 25.81$, $P=.01$, partial eta squared = .892) than those who scored lower, and higher risk magnitude ratings were given by those who scored higher on neuroticism ($F(1, 3340) = 49.97$, $P=.005$, partial eta squared = .942) and lower on agreeableness ($F(1, 3340) = 12.36$, $P=.04$, partial eta squared = .803). The effects of openness on risk magnitude and of neuroticism and agreeableness on believability were not significant.
